# Engaging Veterans in Identifying Key Elements of Environmental Cleaning: The Patient Perspective

**DOI:** 10.1017/ash.2021.120

**Published:** 2021-07-29

**Authors:** Kelsey Baubie, Linda McKinley, Julie Keating, Rosemary Bartel

## Abstract

**Background:** Contaminated surfaces in healthcare settings contribute to the transmission of pathogens. Environmental cleaning and disinfection are important for preventing pathogen transmission and reducing healthcare-associated infections (HAIs). Hospital cleanliness plays a large role in patient perception of the healthcare setting and, consequently, of patient satisfaction. However, patient perceptions of environmental cleaning remain unclear. To engage patients as part of achieving patient-centered care, we undertook a qualitative study to examine patient perspectives on environmental cleaning and disinfection in healthcare settings. **Methods:** We conducted semistructured qualitative interviews with 14 hospitalized patients at a large midwestern Veterans’ Administration hospital. Interviews were audio recorded, professionally transcribed verbatim, summarized in a “key domains” template developed by the research team, then coded for emerging themes. **Results:** Patients reported feeling satisfied with hospital cleanliness and especially the daily cleaning they observed while hospitalized. Cleaning activities highlighted included mopping and disinfecting high-touch surfaces, bathrooms, and floors. Despite this overall positive response, some patients expressed worries of being “in the way” or burdensome if they were in their rooms while staff were cleaning. One interviewee stated, “It’s easier for them if there isn’t a patient in [the room] … it’s hard to do any endeavor when you’ve got a complete stranger watching you.” Patients also acknowledged the importance of careful cleaning, especially during the COVID-19 crisis; “It’s got to be something you take seriously, especially during this pandemic.” Some patients spoke of the relationship which can develop between environmental services staff during daily hospital room cleaning. **Conclusions:** Patient perceptions of environmental cleaning are important to understand and incorporate into clinical practice. Overall, patients felt that their environments were clean, and they expressed confidence in the staff’s work. Interviewees additionally spoke of their own self-efficacy, saying they try to clean up after themselves and would feel comfortable speaking up if something needed to be cleaned. However, some patients acknowledged feeling burdensome to the environmental services staff if patients were present in rooms while staff cleaned. Cleaning activities may become more patient-centric if they are better planned (eg, while patient is out of the room) or based on patient preferences on time of day.

**Funding:** No

**Disclosures:** None

